# Access to electronic health records by care setting and provider type: perceptions of cancer care providers in Ontario, Canada

**DOI:** 10.1186/1472-6947-9-38

**Published:** 2009-08-10

**Authors:** Margo C Orchard, Mark J Dobrow, Lawrence Paszat, Hedy Jiang, Patrick Brown

**Affiliations:** 1 Department of Health Policy, Management & Evaluation, University of Toronto, 1 University Avenue, Suite 300, Toronto, Ontario, M5J 2P1, Canada; 2 Cancer Services & Policy Research Unit, Cancer Care Ontario, 620 University Avenue, Toronto, Ontario, M5G 2L7, Canada; 3 Institute for Clinical and Evaluative Sciences, 2075 Bayview Avenue, Room G1-46, Toronto, Ontario M4N 3M5, Canada; 4 Population Studies and Surveillance, Cancer Care Ontario, 620 University Avenue, Toronto, Ontario, M5G 2L7, Canada; 5 Dalla Lana School of Public Health, University of Toronto, 155 College Street, Toronto, ON, M5T 3M7, Canada

## Abstract

**Background:**

The use of electronic health records (EHRs) to support the organization and delivery of healthcare is evolving rapidly. However, little is known regarding potential variation in access to EHRs by provider type or care setting. This paper reports on observed variation in the perceptions of access to EHRs by a wide range of cancer care providers covering diverse cancer care settings in Ontario, Canada.

**Methods:**

Perspectives were sought regarding EHR access and health record completeness for cancer patients as part of an internet survey of 5663 cancer care providers and administrators in Ontario. Data were analyzed using a multilevel logistic regression model. Provider type, location of work, and access to computer or internet were included as covariates in the model.

**Results:**

A total of 1997 of 5663 (35%) valid responses were collected. Focusing on data from cancer care providers (N = 1247), significant variation in EHR access and health record completeness was observed between provider types, location of work, and level of computer access. Providers who worked in community hospitals were half as likely as those who worked in teaching hospitals to have access to their patients' EHRs (OR 0.45 95% CI: 0.24–0.85, p < 0.05) and were six times less likely to have access to other organizations' EHRs (OR 0.15 95% CI: 0.02–1.00, p < 0.05). Compared to surgeons, nurses (OR 3.47 95% CI: 1.80–6.68, p < 0.05), radiation therapists/physicists (OR 7.86 95% CI: 2.54–25.34, p < 0.05), and other clinicians (OR 4.92 95% CI: 2.15–11.27, p < 0.05) were more likely to report good access to their organization's EHRs.

**Conclusion:**

Variability in access across different provider groups, organization types, and geographic locations illustrates the fragmented nature of EHR adoption in the cancer system. Along with focusing on technological aspects of EHR adoption within organizations, it is essential that there is cross-organizational and cross-provider access to EHRs to ensure patient continuity of care, system efficiency, and high quality care.

## Background

The aim of this paper was to examine perceptions of access to electronic health records (EHRs) by different types of cancer care providers in a wide range of care settings. The real-world perspectives of an array of cancer care providers, including physicians, nurses, case managers, radiation therapists, and dieticians, provide insights on factors that may influence adoption or use of EHRs and the integration and continuity of care. The data focus on access within and across organizations and the completeness of health records.

In the context of this research, Electronic Health Records (EHR) refer broadly to the use of health information technology (HIT), including patient health information and data, clinical decision support, results management and central data repositories, and order entry management technologies such as computerized physician order entry (CPOE), to support the organization and delivery of care [1-3]. The use of EHRs to support healthcare systems internationally is evolving and expanding. It has been suggested that these systems increase efficiency, improve patient safety, and are associated with improved health benefits [1] and more efficient use of physician time [4]

HIT is being used to improve health system efficiency, integration, and the patient experience in developed nations across the world [5,6] Germany, the United Kingdom, Norway, and Australia have all established or initiated major HIT programs with varying levels of clinician involvement [5,6] In contrast, Canada has been slower to adopt HIT and EHRs. The 2006 Commonwealth Fund International Health Policy Survey of Primary Care Physicians shows Canada falling behind the UK, Germany, the Netherlands, Australia, New Zealand, and the United States in the use of information systems to support patient care [6]. Canadian primary care physicians report lower rates of EHR use for communication both within and outside of their practices. Compared to these other countries, Canadian primary care physicians also report lower rates of electronic test ordering and prescribing, reduced electronic access to patient test results and hospital records, and lower rates of the use of computerized prompts to indicate drug safety concerns, test results, and reminders [6]. Furthermore, only 4% of Canadian primary care practices have the technological capacities that allow for advanced clinical information functions [7], thus leaving Canadian primary care physicians poorly equipped to improve the management of patients with chronic disease [6]. Patient surveys echo these results. For example, only 9% of Canadian patients can communicate with their primary care doctors by email, compared to 20% in the US and 22% in New Zealand [8]. Although the Commonwealth Fund survey describes primary care physician adoption of the EHR, less is known about Canada's adoption of EHR and information technologies in other domains of healthcare, and by other types of providers (such as nurses and pharmacists).

The province of Ontario represents one of the world's largest publicly funded and managed health systems (e.g., $40 billion annual budget). Despite local advances in EHR technology such as the Laboratory Information System, emergency room access to medication profiles, the Wait Time Information System [9], diagnostic imaging systems, and drug information systems, 26% of physician specialists in Ontario report no internet access in their main patient care setting, only 30% have access to their patients' EHRs, and just 20% have an electronic interface to other external systems for accessing or sharing patient information [10]. There is considerable variation among Ontario hospitals in the use of information technologies, with a trend towards higher rates of adoption in academic institutions compared to community hospitals [11]. However, this trend does not pertain to all types or settings of care. For example, Lapinsky and colleagues conducted a survey on intensive care units (ICUs) across Ontario and found no relationship between ICU size or university affiliation and IT availability [12]. These inconsistent trends reflect the complexity of issues influencing access to EHRs by types and settings of care, supporting the need for more specific analyses of access to EHRs.

The use of EHRs in cancer systems is a case in point. Cancer systems are a microcosm of broader healthcare systems. They represent a diverse and complex range of delivery system entities, from academic to community hospitals, and from comprehensive cancer centres to community-based home care. Cancer patients receive care from a variety of providers, including medical oncologists, radiation oncologists, surgeons, radiation therapists, social workers, dieticians, and nurses. These providers work in many different types of environments and cancer patients cross boundaries both within care settings (e.g., from surgery, to pathology, to the systemic treatment suite, to the radiation treatment facility) and between care settings (from the home, to a local hospital, to a comprehensive cancer centre). In Ontario, cancer services are coordinated centrally by the provincial cancer agency (Cancer Care Ontario), organized regionally by 14 regional cancer programs, and delivered locally by more than 80 hospitals, 14 Community Care Access Centres and numerous organizations providing community care services [5].

EHRs for cancer services can include computerized physician order entry (CPOE), diagnostic information systems, scheduling systems, and patient portals that can play a critical role in improving service integration and continuity of care [13,14]. While Ontario currently lacks system-wide EHR infrastructure or capabilities, relying on a number of institution-specific systems, the cancer system is developing tools and information systems to help increase the use of EHRs in the province. For example, 62% of all systemic treatment visits are supported by CPOE and 43% of lung cancer patients complete a web-based symptom screening tool when they visit the cancer centre [15]. However, with the complex context for cancer services, little is known about cancer care providers' access to organizations' existing EHRs or the completeness of these EHRs across the many care settings in the province.

Along with evidence that an EHR improves efficiency, patient safety, and health outcomes [1], there is also evidence that an EHR can improve functional and clinical integration [16,17] Having an EHR that is accessible to providers across multiple facilities (including access to online appointment scheduling, and HIT innovations to improve access to information) is an important part of improving cancer system performance [18]. Although this requires the participation of more than just physicians, the collaborative multi-professional requirements for EHRs are often overlooked [19]. For example, CPOE involves nurses, pharmacists, physiotherapists, radiologists and laboratory technicians [20] and requires that each of these groups have access to the technology before it can be used efficiently. Although communication should be bi-directional, a study of Dutch physicians, nurses, and pharmacists found that these groups only used CPOE for one-way communication [19]. In reality, a physician or nurse enters the drug order, a pharmacist checks the dose and processes the order, and then returns a medication sheet and the prepared dose to the nurse for dispensing [20]. CPOE allows for centralized decision-making and streamlined communication between groups [21], however unless all of the providers involved have access to the technology, the benefits of CPOE and other EHR-supported tools cannot be achieved.

This research aimed to determine the real-world perspectives of an array of cancer care providers in a wide range of care settings. More specifically, we wanted to explore the relationship between provider type, location of work, and level of access to a computer/the internet, and cancer care providers' perceptions of health record completeness and access to patient EHRs (both within and across organizations).

## Methods

As part of a larger survey, cancer care providers and administrators were surveyed for their perceptions regarding access to EHRs, access to computers, and access to the internet. The Cancer Services Integration (CSI) Survey is an annual internet-based survey of cancer care providers and administrators representing all 14 regional cancer programs in Ontario [22,23] Regional cancer programs in Ontario are responsible for coordinating cancer services across multiple service provider organizations within geographically defined and mutually exclusive regions. A list of cancer care providers and administrators was compiled using the Canadian Medical Directory, Cancer Care Ontario's list of clinical and administrative cancer program leaders and direct contact with individual hospitals and Community Care Access Centres. For the 2008 survey, all identified cancer care providers and administrators in the province (n = 5663) were sent an email invitation with a web link to the online survey. Up to three email reminders were sent to non-responders over a three week period. Survey responses were captured electronically at the time of response and were subsequently downloaded for analysis. Only data for cancer care providers (N = 1247) were included in the analysis.

The three questions considered in the analysis were: 1) I have access to *my *organization's electronic health records for the cancer patients that I am responsible for; 2) I have access to *other *organizations' electronic health records for the cancer patients that I am responsible for; 3) Health records (either paper or electronic) for the cancer patients I am responsible for are usually complete.

Descriptive summary statistics were tabulated for respondent characteristics. For univariate comparisons, responses for the three questions were dichotomized as positive ('strongly agree', or 'agree' from Likert scale) or negative (all other valid responses). Proportions were calculated for all positive and negative responses for each question.

A multilevel logistic regression model was fit for each question to assess the marginal contribution of each variable. A random effect term was included at the region level to account for clustering of respondents within regional cancer programs. Consideration of respondent clustering within regions was important as regional cancer programs were at varying stages of development at the time of the survey. A separate model was created for each of the three questions as follows:

Y_ij _is the response from respondent *j *in region *i*, *X*_*i*j _is a vector of covariates for this respondent, *β *is a vector of regression coefficients, *U*_i _is the region-level random effect, and *σ*^2 ^is the variance of this random effect. Missing and 'not applicable' responses were excluded. Self-identified respondent position/role (profession), primary location of work, and access to computer or internet were included as covariates in the model representing possible confounders in the *X*_*i*j _term. The reference groups for each variable were assigned as follows: position/role = surgeons; primary location of work = teaching hospital with cancer centre; computer access = access to computer and the internet. These reference groups were generally chosen to be the largest groups, to minimize correlation between estimated parameters. For profession, surgeons were chosen as the reference group because they work in more varied care settings compared with the other professions. Odds ratios and their 95% confidence intervals were calculated for each variable and a p-value < 0.05 was considered statistically significant.

The software SAS 9.1 (SAS Institute, North Carolina, US) was used for performing descriptive and univariate analyses. The SAS procedure GLIMMIX was used for model fitting and estimation; random effects were normally distributed, a Dual Quasi-Newton optimization technique was used; and the estimation method was residual log pseudo-likelihood [24].

## Results

### Respondent characteristics

Of 5663 email invitations distributed, a total of 1997 (35%) valid responses were collected. Because we wanted to capture the perspectives of cancer care providers, the administrator responses (n = 750) were not included in the analysis.

Overall, 23% of physicians and 36% of other clinicians participated in the survey. A similar pattern of response rates was seen in the 2007 CSI survey [22]. The distribution of demographic characteristics of respondents included in the sub-analysis is shown in Table 1. Non-responders and responders were comparable on two key characteristics, region and profession. Regionally, non-responders and responders differed minimally, while non-responders were more likely to be physicians. Questions 1, 2, and 3 had 10%, 11%, and 9% missing data respectively.

**Table 1:** Distribution of respondent characteristics

Participant Breakdown		N	%
**Sex**			

	Female	807	65%

	Male	433	35%

**Age**			

	60 years and older	95	8%
	
	50–59 years	420	34%
	
	40–49 years	400	32%
	
	under 40 years	324	26%

**Participant Group**			

	Surgeons (surgical oncologist, general surgeon, gynecologist, urologist, thoracic)	162	13%
	
	Medical oncologists	55	4%
	
	Radiation oncologists	63	5%
	
	Other physicians (general practitioner in oncology, gastroenterologist, hematologist, hospitalist, palliative care physician, pathologist, radiologist, respirologist)	247	20%
	
	Nurses (advanced practice, chemotherapy, clinical trials, inpatient oncology, Ontario Breast Screening Program, and primary care nurses)	328	26%
	
	Radiation therapists/physicists	171	14%
	
	Other clinicians (pharmacist, dietician, social worker)	176	14%
	
	Case managers	43	3%

**Primary location of work**			

	Teaching hospital (with a cancer centre)	611	49%
	
	Teaching hospital (without a cancer centre)	59	5%
	
	Community hospital (with a cancer centre)	267	21%
	
	Community hospital (without a cancer centre)	212	17%
	
	Other location	52	4%
	
	Community-based care	46	4%

**Total**		**1247**	

### Question-specific responses

A summary of positive responses for each question is shown in Table 2. Overall, 80% of respondents indicated that they had good access to their organization's EHRs ('agree' or 'strongly agree'). In contrast, participants reported unfavourable access to other organizations' EHRs (22% reporting good access). Approximately half of respondents indicated that their organization's paper or electronic health records were usually complete (Table 2).

**Table 2:** Summary of question responses

	Percent responding favourably (agree or strongly agree)					
	QUESTION 1: I have access to my organization's electronic health records		QUESTION 2: I have access to other organizations' electronic health records		QUESTION 3: Health records (either paper or electronic) are usually complete	

	N	%	N	%	N	%

**All respondents**	994	80%	275	22%	583	47%

						

**Computer/Internet Access**						
Computer and internet	755	89%	215	25%	463	55%
computer but no internet	40	87%	15	33%	20	43%
no computer	175	71%	39	16%	86	35%

						

**Participant group:**						
Surgeons	117	72%	26	16%	75	46%
Medical oncologists	49	89%	19	35%	29	53%
Radiation oncologists	54	86%	16	25%	22	35%
Other physicians	183	74%	61	25%	95	38%
Nurses	268	82%	78	24%	169	52%
Radiation therapists/physicists	137	80%	37	22%	79	46%
Case managers	31	72%	7	16%	16	37%
Other clinicians	155	88%	31	18%	97	55%

						

**Primary work location**						
Teaching hospital with cancer centre	508	83%	145	24%	263	43%
Teaching hospital without cancer centre	41	69%	12	20%	19	32%
Community hospital with cancer centre	220	82%	150	25%	150	56%
Community hospital without cancer centre	165	78%	35	17%	106	50%
Community Care Access Centre	33	72%	5	11%	20	43%
Other location	27	52%	12	23%	25	48%

### Multilevel logistic regression model

Table 3 summarises the results from the mixed model fit to each of the three questions. The respondent's self-identified profession and primary work location had varying impact on the proportion of individuals most likely to respond favourably to the three questions. To validate the findings, we analyzed respondents' access to a computer and the internet which we anticipated would be related to favourable EHR access. As anticipated, having access to a computer or the internet was associated with significantly better odds of reporting good access to EHRs (both within and outside their organization) and complete health records, thus providing a face validity check.

**Table 3:** Results of multilevel logistic regression model

	Parameter Estimates					
	**I have access to my organization's electronic health records**		**I have access to other organizations' electronic health records**		**Health records (either paper or electronic) are usually complete**	
	
	**OR (95%CI)**	**p**	**OR (95%CI)**	**p**	**OR (95%CI)**	**p**

**Computer/Internet Access**						

Computer and internet	1.00	-	1.00	-	1.00	-

Computer but no internet	0.80 (0.26, 2.47)	0.699	1.43 (0.73, 2.80)	0.295	0.53 (0.28, 1.00)	0.050

No computer	0.29 (0.19, 0.45)	< .001	0.56 (0.38, 0.83)	0.004	0.40 (0.30, 0.55)	< .001

**Participant group:**						
Surgeons	1.00	-	1.00	-	1.00	-
Medical oncologists	2.75 (0.88, 8.61)	0.082	2.36 (1.14, 4.87)	0.021	1.02 (0.54, 1.95)	0.946
Radiation oncologists	1.30 (0.50, 3.37)	0.591	1.55 (0.74, 3.26)	0.249	0.59 (0.31, 1.13)	0.114
Other physicians	1.28 (0.73, 2.25)	0.385	1.80 (1.05, 3.07)	0.032	0.69 (0.45, 1.07)	0.099
Nurses	3.47 (1.80, 6.68)	< .001	1.53 (0.91, 2.59)	0.112	1.26 (0.82, 1.92)	0.288
Radiation therapists/physicists	7.86 (2.54, 24.34)	< .001	1.46 (0.79, 2.69)	0.227	1.27 (0.77, 2.10)	0.349
Case managers	4.13 (0.17, 102.4)	0.387	5.18 (0.84, 31.95)	0.077	0.25 (0.06, 1.04)	0.057
Other clinicians	4.92 (2.15, 11.27)	< .001	0.98 (0.53, 1.79)	0.939	1.27 (0.79, 2.04)	0.317

**Primary work location**						
Teaching hospital with cancer centre	1.00	-	1.00	-	1.00	-
Teaching hospital without cancer centre	0.72 (0.28, 1.83)	0.491	1.02 (0.50, 2.08)	0.968	0.80 (0.42, 1.53)	0.505
Community hospital with cancer centre	0.45 (0.24, 0.85)	0.014	0.94 (0.62, 1.42)	0.761	1.60 (1.13, 2.28)	0.009
Community hospital without cancer centre	0.55 (0.30, 1.01)	0.054	0.62 (0.39, 0.99)	0.045	1.47 (1.01, 2.13)	0.046
Community-based care	1.09 (0.04, 26.58)	0.960	0.15 (0.02, 0.10)	0.050	3.81 (0.95, 15.35)	0.060
Other location	0.29 (0.12, 0.71)	0.006	0.92 (0.42, 2.02)	0.837	2.05 (1.04, 4.03)	0.038

**Region-level variance parameter**	Estimate (standard error)		Estimate (standard error)		Estimate (standard error)	
	0.58 (0.34)		0.15 (0.10)		0.05 (0.04)	

#### Perceptions of access to own organization's EHRs

Compared to surgeons, nurses (OR 3.47 95% CI: 1.80–6.68, p < 0.05), radiation therapists/physicists (OR 7.86 95% CI: 2.54–25.34, p < 0.05), and other clinicians (OR 4.92 95% CI: 2.15–11.27, p < 0.05) were significantly more likely to report good access to their organization's EHRs. There was no significant difference between surgeons and medical oncologists, radiation oncologists, other physicians, or case managers. Respondents who did not report good access to a computer were statistically less likely to report good access to their own organization's EHRs compared to individuals who reported having access to a computer and the internet (OR 0.29 95% CI: 0.19–0.45, p < 0.05). In terms of location of work, individuals who worked in community hospitals were half as likely as respondents who worked in teaching hospitals with a cancer centre to report good access to their own organization's EHRs (OR 0.45 95% CI: 0.24–0.85, p < 0.05).

#### Perceptions of access to other organizations' EHRs

Medical oncologists were more than twice as likely as surgeons to report good access to other organizations' EHRs (OR 2.36 95% CI: 1.14–4.87, p < 0.05) while other physicians (such as gastroenterologists, hematologists, and radiologists) were almost two times as likely as surgeons (OR 1.80, 95% CI: 1.05–3.07, p < 0.05). Although not statistically significant, radiation oncologists, nurses, and case managers all reported higher odds of having access to other organizations' EHRs. Respondents who did not have good access to a computer were half as likely to report good access to other organizations' EHRs compared to individuals who reported good access to a computer and the internet (OR 0.56 95% CI: 0.38–0.83, p < 0.05).

In terms of location of work, individuals who worked in community-based care settings were six times less likely than respondents who worked in teaching hospitals with a comprehensive cancer centre to report good access to other organization's EHRs (OR 0.15 95% CI: 0.02–1.00, p < 0.05). Similarly, respondents in community hospitals that did not contain a comprehensive cancer centre were 1.5 times less likely than respondents in teaching hospitals with comprehensive cancer centres to report good access to other organizations' EHRs (OR 0.62 95% CI: 0.39–0.99, p < 0.05).

#### Perceptions of health record completeness (either paper or electronic)

Respondent profession did not influence perception of health record completeness. However, good access to a computer and the internet was associated with better health record completeness. Respondents who did not have good access to a computer or the internet were 2.5 times less likely to report that their organization's health records were complete than respondents who had good access to both a computer and the internet (OR 0.40 95% CI: 0.30–0.55, p < 0.001). Respondents who did not have good access to the internet (but did have good access to a computer) were two times less likely to report that their health records were complete (OR 0.53 95% CI: 0.28–1.0, p = 0.05). Perceived completeness of health records also varied depending on the respondent's location of work. Respondents working outside of teaching hospitals (such as community-based care settings or community hospitals) were more likely to report that their health records were complete. For example, respondents in community hospitals were 1.5 times as likely to report that their health records were usually complete compared to respondents in teaching hospitals with a comprehensive cancer centre (OR 1.47, 95% CI 1.01–2.13, p < 0.05).

## Discussion

Using a multilevel model, this study demonstrates statistically significant variability in access to EHRs across the Ontario cancer system. Cancer care providers report variation in access to EHRs both within and across their organizations. For example, although 80% of respondents reported that they had good access to EHRs at their own organization, only 22% reported having good access to other organizations' EHRs. Cancer care providers also reported variation in perceived access to EHRs and in the completeness of health records across provider and organization types.

### Access to a computer and the internet

Respondents that did not report good access to a computer or the internet reported poorer access to EHRs and less complete health records than those who reported good access to both a computer and the internet. This provides face validity and is an indication that basic information technology access is associated with more complete health records. This also suggests that investments in HIT could result in better and more complete patient health records.

### Variation by provider type

Although good access to computers and the internet is necessary to support the application of EHRs within and across organizations, these data indicate that there is varying access to EHRs across provider types, even for providers working within the same institution. For example, radiation therapists are six times more likely to report good access to their patients' EHRs than radiation oncologists. Because in Ontario, they are limited to working at comprehensive cancer centres with radiation treatment equipment/facilities, both radiation oncologists and radiation therapists interact with the same types of patients in the same settings. Although it is possible that radiation oncologists could delegate computer-related tasks to radiation therapists or nurses, therefore having potentially less need to access EHRs, the survey question addressed *perceived access *to EHRs (rather than *actual use*). This means that respondents who did not actually use EHRs (e.g., because they were delegating tasks to others) could still respond favourably to the question if they perceived the EHR system to be functioning well. Therefore, it is possible that the EHR needs of radiation oncologists and radiation therapists differ and that their perceptions of access actually refer to specific EHR/HIT systems which these data do not reveal. In general, non-physician providers report better access to their own organization's EHRs than physicians. This could be due to different applications of EHRs. For example, radiation therapists may only use EHRs within their unit of the hospital, whereas radiation oncologists may need access to EHRs for patients referred from community care settings, particularly following surgery or systemic treatment, which are provided in a wider range of health care settings.

There is also variation among provider types in terms of perceived access to EHRs outside of their organizations. For example, medical oncologists and other physicians (such as gastroenterologists, hematologists, and radiologists) were approximately twice as likely as surgeons to report good access to EHRs outside of their organization. It is possible that physicians such as medical oncologists interact more directly with patients in other hospitals (such as systemic treatment outreach clinics), and so have a more direct need to access the EHRs of these patients.

In summary, nurses, radiation therapists/physicists and other clinicians were more likely than surgeons to report good access to EHRs within their organization, whereas medical oncologists and other physicians were more likely than surgeons to report good access to other organizations' EHRs. Because individual providers have different needs for and applications of EHRs, some variation between provider types is expected. However, it is unclear how much variation is appropriate. It is clear that EHRs can increase collaboration among care teams [25] but before they can contribute to increased effectiveness and efficiency, further consideration is needed to balance accessibility and privacy concerns. If placed in the wrong context, EHRs could have little benefit in improving quality of care, while reducing privacy of patient information [26]. Likewise, if not all of the relevant team members have access to EHRs, this could have a negative impact on integration and continuity of care.

### Variation by location of work

Providers working in community hospitals and those providing community-based care report poorer access to EHRs both within their organization and across organizations. Similar trends have been reported in administrator-based surveys [11]. It has been suggested that larger teaching hospitals have better HIT support and in-house capabilities to share and exchange data [27]. Lower HIT- and EHR-capabilities at community-based organizations may promulgate fragmented care and could have implications for continuity of care and system efficiency.

The results also indicate that it is possible for cancer care providers to have good access to EHRs internally, but at the same time have poor access externally. For example, individuals who worked in community-based care organizations were as likely to report good access to their internal EHRs as those in teaching hospitals, but were significantly less likely to report good access to EHRs outside of their organization. Because home care requires interaction with the acute and outpatient care sectors, this lack of external connectivity could have implications for integrated service delivery and efficiency. This also indicates that even though an organization may be "wired" and have internal access to EHRs, it may not have access to the patient health records from other organizations, even if these organizations are also electronic-based. Reasons for these cross-institutional barriers to the sharing of EHRs could include privacy issues and software heterogeneity or lack of cooperation due to competitive or political issues. It should be noted that despite the fact that community hospitals and community-based care providers are less likely to report good cross-institutional access to EHRs, they are more likely to report that their health records are complete than teaching hospitals. This could partly be due to variations in perception and expectations, as well as the relatively smaller size and complexity of cases at these centres compared to teaching hospitals.

### Limitations

A number of limitations associated with this research should be considered. First, the response rate for this study was low. However, the response rates obtained are common in surveys of clinicians [28]. In addition, the survey essentially represented the full population, rather than a sub-sample, of cancer care providers in Ontario. Furthermore there was little difference in the distribution of respondents and non-respondents in terms of region, profession, and organization type.

A second limitation of the research is the delivery method. The internet survey delivery method required that respondents have access to a computer, thus potentially biasing the results. However, in our numerous interactions with provider organizations during the survey development phase of the project, concerns that specific provider groups would be disenfranchised due to the survey method were not expressed. In addition, in the 2007 CSI survey, less than 3% of the original sample was excluded because of lack of email or computer access.

A third limitation is that there was potential for respondents to misinterpret the survey questions. For example, the questions do not differentiate between *perceived *access and *actual *access to EHRs, nor do they differentiate between easy and difficult access. Likert scale questions are based on perception, and their interpretation is complex. Although it is possible that some provider types may have interpreted these questions differently than others, problems of interpretation of survey questions are an inevitable aspect of survey research. In pilot testing of the survey questions, respondents did not report any difficulty understanding or answering these questions.

Finally, there are two potential limitations regarding the generalizability of our findings to other health system contexts. Because this research is based on providers working in Ontario, Canada, a publicly financed healthcare system, factors influencing the generalizability to other types of health care systems must be considered. However, many aspects of health services integration, including informational continuity, have been shown to be consistent across system types, thereby reducing this concern [22]. The focus on cancer care and the exclusion of primary care providers may also limit the generalizability of the findings. However, there has been considerable research exploring primary care access to EHRs [6,8]. and the current research fills a gap in identifying access to EHRs by a range of providers across the cancer system.

## Conclusion

This study explored the perceptions of EHR access by a wide range of cancer care providers covering diverse cancer care settings. Although cancer care providers in Ontario generally report good access to EHRs within their organizations, gaps remain in access to EHRs from other organizations. This emphasizes the fragmented nature of EHR adoption and cross-communication in cancer systems. Along with focusing on EHR implementation within isolated organizations, it is essential that there is cross-institutional and cross-provider communication to ensure patient continuity of care and system efficiency. Similarly, reported completeness of health records, a possible indicator of the quality of EHRs, varies across organization and provider-type. Organizations and providers that report poorer access to EHRs also report poorer completeness of health records, indicating that there may be an association between access to EHRs and the completeness of health records.

There are a number of potential solutions that could help to ensure consistent and coherent EHR adoption. For example, a health system could begin developing interoperability standards for consistent and compatible EHR formats. A similar process of developing standards for EHR implementation is underway in Europe [29]. The ability for providers to communicate with a common EHR system is especially important for cancer care, as patients frequently cross between hospitals and other care settings. Health systems should also adopt policy approaches to integrate EHR development. For example, the European Union is beginning to promote electronic health action plans and research [30]. Recently, Ontario has developed a coordinated e-Health strategy and is focusing on using HIT to improve patient care, access, and safety [31]. Similarly, Canada Health Infoway is working at the national level to accelerate the use of EHRs across the country [32]. By providing political pressure for consistent and standard EHR adoption, health systems will be better equipped to communicate both within and across organizations. Finally, further research is required to identify provider-specific gaps in EHR access and factors influencing cross-organization EHR access.

This study provides a baseline view of EHR access for cancer care providers in a large healthcare system. There are a number of challenges to the adoption of EHRs [33] but by understanding the current differences in access by provider type and care setting, we can begin to explore barriers to implementation and solutions to improve continuity of care for patients. This knowledge will be essential for exploring current uptake, applications, and gaps in the use of EHRs across healthcare systems.

## Competing interests

The authors declare that they have no competing interests.

## Authors' contributions

MJD and LP helped to conceptualize the study. MJD, LP and MO participated in the design and conduct of the survey. PB and HJ performed the statistical analyses. MO wrote the initial draft of the manuscript, which all authors reviewed and provided feedback. All authors read and approved the final manuscript.

## Pre-publication history

The pre-publication history for this paper can be accessed here:

http://www.biomedcentral.com/1472-6947/9/38/prepub
